# The development of a current events and dialogue forum at a large U.S. academic medical center

**DOI:** 10.1007/s40037-021-00651-2

**Published:** 2021-01-29

**Authors:** Andrew W. Kuhn, Eriny S. Hanna, Varun K. Menon, Ryan T. Jarrett, VC2 Committee, Mobolanle Adebesin, Jackie Antoun, Joshua Bland, Rebekka DePew, Demetra Hufnagel, Elishama Kanu, Karampreet Kaur, Christian Nguyen, Daniel Pereira, Maxwell Roeske, Samuel Trump, Kate L. Payne, André L. Churchwell

**Affiliations:** 1 Vanderbilt University School of Medicine, Nashville, TN USA; 2 Department of Biostatistics, Vanderbilt University School of Medicine, Nashville, TN USA; 3 Center for Biomedical Ethics and Society, Vanderbilt University Medical Center, Nashville, TN USA; 4 Office for Diversity Affairs, Vanderbilt University Medical Center, Nashville, TN USA

**Keywords:** Empathy, Current events, Dialogue, Community

## Abstract

**Background:**

The Vanderbilt Community Circle (VC2) was designed to provide all faculty, staff, and students within the entire Vanderbilt University Medical Center community a dedicated venue to discuss current events and ongoing societal issues.

**Approach:**

During the 2017–18 academic year, four VC2 events were held on: “Race, identity, and conflict in America,” “Gun violence in America,” “Gender in the workplace,” and “Immigration in America.” Facilitators guided participants to share their views and perspectives on these matters with pre-developed open-ended questions. Attendees started discussions in small groups and then eventually combined into a large one. Pre- and post-event surveys were administered to measure the program’s effectiveness.

**Evaluation:**

One-hundred and twenty-four participants were included, 75 of whom completed both the pre- and post-event surveys. Sixty-four of the 75 (85%) agreed or strongly agreed that “multiple perspectives and opinions were represented” and 73% felt that their “own perspective was broadened on the issue.” Most (89%) believed that the format and setting of the event was conducive to dialogue and discussion, and almost all (91%) reported that they would attend a similar event in the future. Groningen Reflection Ability Scale scores were high before (94 [25th–75th: 88–99]) and remained high after the events (93 [25th–75th: 88–93.3], *p* > 0.05).

**Reflection:**

We successfully implemented a medical center-wide, recurring current events and dialogue forum in hopes of increasing reflection, unity, and understanding across our own community.

**Supplementary Information:**

The online version of this article (10.1007/s40037-021-00651-2) contains supplementary material, which is available to authorized users.

## Background and need for innovation

The current landscape of healthcare delivery in large academic medical centers requires successful interdisciplinary care in order to maximize outcome measures most relevant to patients and the system in which they are served [1–3]. Successful interdisciplinary care hinges not only on the health and well-being of each of its individual members, but also on individuals being able to openly communicate, connect, reflect, and understand each other’s feelings and perspectives.

Recent political and societal changes have exemplified the effects that current events can have on the emotional health and well-being of individuals. For instance, following the previous U.S. presidential election in 2016, healthcare professionals felt there was increased racial hostility, community-level prejudice, animosity towards immigrants, and concerns about reductions in social and health services [4]. These sentiments are not unique to the U.S. and extend globally. Importantly, feelings such as these may cause division or tension within an organization.

## Goal of innovation

The Vanderbilt Community Circle (VC2) is an initiative developed to provide faculty, staff, and students of the entire Vanderbilt University Medical Center (VUMC) community with a dedicated venue to discuss current events and ongoing societal issues within an open and inclusive setting. The *a priori *goal of VC2 was not to change individuals’ opinions, but rather to broaden perspectives through open dialogue and communication. Our hope was that of increasing reflection, unity, and understanding across our own medical center community.

## Steps taken for development and implementation of innovation

### VC2 committee and leadership

In 2016, a committee was formed by two then first-year medical students (AWK, VKM). The committee was designed to include 2–3 individuals from each medical school class (8–12 members total). Individuals apply during their first year and are chosen by group consensus among the standing committee based on peer recommendation, leadership capability and potential, motivation, and interpersonal skills and characteristics. In addition, many of the selected individuals previously had shown a commitment to diversity and inclusion efforts as evidenced by their personal statement and curriculum vitae. The committee is responsible for communicating with multiple departments and deans as well as the student body and organizations to plan and execute VC2 events.

### Timing and structure of VC2 events

VC2 events occur quarterly and are open to the entire VUMC community including faculty, staff, and students. Holding events quarterly allows VC2 to be responsive to recent current events and the needs of the VUMC community while also respecting the scheduling constraints of participants. After careful deliberation, it was decided to hold these events from noon to one in the afternoon to maximize the attendance and diversity of attendees.

Events revolve around a single broad topic such as “Immigration in America” which is generated beforehand by VC2 committee members based on topical current events. To maximize the diversity of backgrounds, opinions, and perspectives at events, advertising has been a crucial component of the VC2 effort. Approximately one month prior to an event, an email advertisement with information, including discussion topic and a reservation link, is disseminated to a wide array of email lists. Emails reach medical students, nurses, and faculty and staff of major VUMC departments. Reminder emails are typically sent two weeks and the day before the event itself.

Each VC2 event is facilitated by one senior faculty member, who is a bioethicist with extensive experience in group discussion and facilitation (KLP), and one rotating VC2 committee member. An “expert” in the pre-selected topic of discussion may also be invited to help facilitate. Ground rules were established with the help of a licensed social worker and counselor from the Employee Assistance Program at VUMC (Tab. 1). Participants are made aware at the beginning of each event that VC2 events are for discussion, not debate. No one is right or wrong and there is no single correct view or perspective. Diversity of thought is welcomed and encouraged.

**Table 1:** Ground rules established for VC2 events

Respect others when they are talking
Listen and hear what others are saying. Let them finish talking. Think before you react
Be conscious of body language and nonverbal responses
The goal is not necessarily to agree but rather to gain a deeper understanding
Talk about yourself and your own experiences
Refrain from personal attacks and judgment. Focus on ideas
Participate as you feel comfortable
Treat this group as a private conversation. Do not repeat elsewhere what is said during the discussion
Remember that while this process is not intended to be therapeutic, it may be for some
Remove indicators of individual titles or positions, including name badges with credentials

Facilitators initiate discussion with a single question, for example, “What words come to mind when you hear the word ‘immigrant’?” Participants begin in small groups (approximately five to eight individuals) and then slowly combine with other groups until the discussion is open to the entire floor. Facilitators have several other pre-written questions to guide the discussion as necessary in order to promote healthy and respectful dialogue with diverse perspectives. These questions are designed to be neutral and unbiased and remain open-ended to generate discussion. Should the discussion become stagnant or polarizing, facilitators guide participants to find common ground with each other.

### Study Design

In an effort to understand the effectiveness and impact of VC2, we administered pre- and post-event surveys to participants attending the four events held during the 2017–2018 academic year. The topics were: “Race, identity, and conflict in America,” “Gun violence in America,” “Gender in the workplace,” and “Immigration in America.”

An invitation to each event was distributed as an email along with the pre-event survey. The pre-event survey included demographic information and attendance of previous VC2 events. It also included the Groningen Reflection Ability Scale (GRAS), an instrument previously used to measure personal reflective ability of medical students [5]. The GRAS is intended to be used as a one-dimensional scale that incorporates three aspects of personal reflection: self-reflection, empathetic reflection, and reflective communication [5, 6].

The post-event survey was emailed immediately after the event, and participants had up to two weeks to complete the first post-event survey. In addition to repeating the GRAS, the post-event survey included questions regarding feedback on the event itself, asking each participant to indicate their level of agreement on a five-point Likert scale with four feedback questions. Participants could also write free-text comments about the event. A second post-event survey was sent after two weeks with only the GRAS. This survey, however, was excluded from the current analysis given the low number of responses. Participants were given the option of receiving a gift card for completing all surveys. This study was considered exempt by the Vanderbilt University Institutional Review Board (#171 484).

### Statistical Analyses

All data were de-identified. Wilcoxon’s signed-rank test was used to evaluate pre- versus post-event survey changes in GRAS scores. For purposes of statistical analyses, demographic characteristics were collapsed into the most frequently reported. Subgroup analyses for changes in GRAS scores included age, gender (male vs. female), race (white vs. non-white), political economic status (conservative, neutral, liberal), education (high school/associate’s, bachelor’s, master’s or doctorate), religion (atheist/agnostic, Christian, other). Responses to the four post-event survey questions were modeled as a function of age, gender, and race (white, Black, other), as well as the respondents’ GRAS score at the post-event survey, and the difference between pre- and post-event GRAS scores. All subgroup analyses were conducted using logistic regression or ordinal logistic regression, depending on the number of viable response categories.

All post-survey questions had relatively few reported values of 1, 2, and 3, (“strongly disagree,”, “disagree,” and “neither agree nor disagree,” respectively) and, consequently, could not be reliably modeled. To account for this, response categories were regrouped. Free-text comments were quantitatively analyzed using an iterative inductive–deductive approach [7].

## Outcomes of innovation

### Participant characteristics

One-hundred and twenty-four participants were included in the analysis, 75 of whom completed both the pre-event and the first post-event survey. The 124 participants were on average 33 (± 13) years old. Most (67.7%) were female. About half (48.4%) were white. Most of the participants were Caucasian or European (46.0%), however other ethnicities were well represented. About half were in medicine (54.8%) and the rest were in nursing, law, or other professions (34.6%). Most (58.9%) had earned a bachelor’s degree as their highest level of education. Christianity was the most represented religion (39.5%), followed by atheism/agnosticism (24.4%). Those who held conservative views, both socially (10.5% vs. 65.3%) and economically (15.3% vs. 51.6%), were less represented than those with liberal views. Around 30% were past attendees. Demographic characteristics can be found in the Electronic Supplementary Material (ESM; see Table 2).

### Groningen Reflection Ability Scale

Median GRAS score pre-event was 94 [25th–75th: 88–99] and post-event was 93 [25th–75th: 88–93.3]. GRAS score was not significantly different between pre-event and post-event surveys with a median difference of 0.99 [95% CI −0.49 to +2.00, *p* = 0.24]. Subgroup analyses did not yield any significant differences between groups (*p* > 0.05).

### Post-event feedback

Eighty-five percent (*n* = 64) of post-forum survey participants agreed or strongly agreed that “multiple perspectives and opinions were represented.” Higher GRAS scores (*OR* = 1.132 [95% CI 1.035–1.237], *p* = 0.0065) and older age (*OR* = 1.087 [95% CI 1.012–1.167], *p* = 0.0222) were significantly associated with stronger agreement with this statement. With the statement, “my own perspective was broadened on the issue,” 73% (*n* = 55) of participants agreed or strongly agreed. Similarly, higher GRAS scores were significantly associated with stronger agreement with this statement (*OR* = 1.096 [95% CI 1.018–1.181], *p* = 0.0151). Eighty-nine percent (*n* = 67) thought that the format and setting of the event were conducive to dialogue and discussion. No significant association with GRAS score or demographic variables was observed for this question. Ninety-one percent (91%) of participants reported they would attend a similar event in the future. A positive association existed between a higher GRAS score and very strong agreement with the statement “I would attend an event like this in the future” (*OR* = 1.368 [95% CI 1.128–1.660], *p* = 0.0015). Statement agreement data can be visualized in ESM (see Fig. 1).

### Free-text commentary

Thirty-two participants in the follow-up survey provided a total of 38 comments. Thirty-seven percent (*n* = 14) of the comments expressed enthusiasm for how the events impacted them. The majority of positive comments regarded broadening of the participant’s perspective or the opportunity the event provided in learning more about the topic. Sixty-three percent (*n* = 24) of comments were feedback statements. Forty-two percent (*n* = 10) of those comments were suggestions about the format or structure of the events. A few participants noted that the table/seating arrangement was not optimal for group discussion. Other participants noted that sitting close to co-workers or peers prevented them from gaining insight into differing perspectives. Thirty-eight percent (*n* = 9) of the comments noted a lack of diversity in perspectives, especially in small group discussion. Some suggested deliberately inviting persons with more conservative views. Twenty-one percent (*n* = 5) of comments noted a desire for deeper discussion and lengthening the duration of the event.

## Critical reflection

We successfully developed a recurring current events and dialogue forum in hopes of increasing reflection, unity, and understanding among members of our own medical center community. The participants surveyed represented a wide range of demographics. With the exception of gender, the demographic make-up of survey participants largely paralleled the population demographics of the surrounding city [8]. It is unclear how the demographic make-up affects the discussion and effectiveness of the forum. For instance, the discussions had at our institution may very well be different from those in other geographical areas or countries. While a “representative sample” of our surrounding population, a more heterogeneous group may have been more effective for the sharing of backgrounds and experiences.

While we did not observe an increase in GRAS scores as a result of the events, the scale may not have been ideal for measuring effectiveness in this setting. Our intervention consisted of one hour of discussion. GRAS has been validated for use only in longer term interventions or for comparing groups at a single time point [9–11]. Additionally, initial GRAS scores were high, suggesting that participants completing the surveys and attending the events were at baseline highly empathetic and reflective. It is also plausible that those with high levels of empathy, as measured by GRAS, self-selected into a VC2 event.

Respondents with higher post-event GRAS scores tended to respond with more agreement to the post-event survey questions addressing the effectiveness of the event, suggesting that the events were perceived to be effective by empathetic participants. Increased age was also associated with stronger agreement with the specific statement regarding broadening of their own perspective due to the event, suggesting a greater perceived impact of the events on older faculty/staff than on younger faculty/staff or students.

The overwhelming majority of participants agreed the forum provided multiple perspectives and opinions and that their own perspectives were broadened. Most participants enjoyed the discussion and reported they would attend a similar event in the future. While the vast majority of respondents believed that multiple perspectives were expressed during the forums, some leaving written feedback noted that they felt that the majority of participants were of the same mindset. Participants with a minority opinion may have hesitated to share their perspective in a group setting. Future efforts should be placed in making participants comfortable with expressing dissenting opinions. These future efforts should be directed toward identifying and personally inviting minority viewpoints. Additionally, participants should be encouraged to sit with attendees they do not know to maximize the opportunity to hear diverse perspectives. If other institutions attempt to replicate this program, certain facets may need to be adjusted to serve the specific institution and its community members (e.g., the 12 pm–1 pm time slot worked best for our institution but may not work as well for others).

## Supplementary Information

Figure 1

Table 2 Participant Demographics (*n* = 124)
