# Supplementary figures and images for: The development of a current events and dialogue forum at a large U.S. academic medical center
Source: Perspect Med Educ. 2021 Jan 29;11(6):371–5. doi: 10.1007/s40037-021-00651-2 (PMC9743831; doi:10.1007/s40037-021-00651-2)

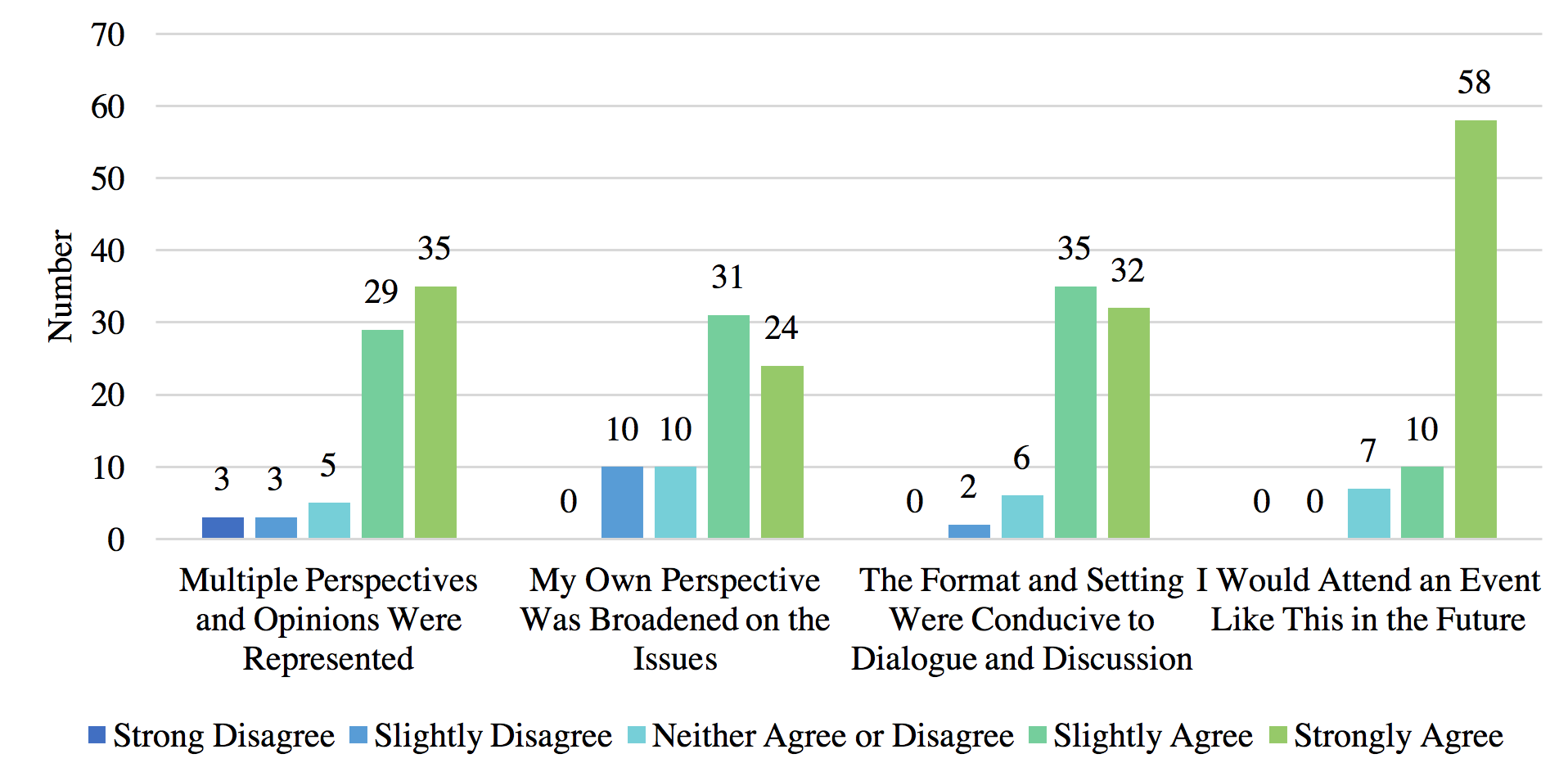

Supplement: Supplementary file 1 — Figure 1 [file 40037_2021_651_MOESM1_ESM.png]
