# Supplementary material for: The development of a current events and dialogue forum at a large U.S. academic medical center
Source: Perspect Med Educ. 2021 Jan 29;11(6):371–5. doi: 10.1007/s40037-021-00651-2 (PMC9743831; doi:10.1007/s40037-021-00651-2)
Supplement: Supplementary file 2 — Table 2 Participant Demographics (n = 124) [file 40037_2021_651_MOESM2_ESM.docx]

**Table 2** Participant Demographics (n=124)

| Age, Mean (SD) | 33 (13) |
| --- | --- |
| Gender, Number (%)  Female  Male | 84 (67.7%)  28 (22.6%) |
| Race, Number (%)  Black  White  Other | 18 (14.5%)  60 (48.4%)  32 (25.8%) |
| Ethnicity, Number (%)  African American  American Indian or Alaskan Native  Arabic or Middle Eastern  Asian  Caucasian or European  Hispanic or Latino  Native Hawaiian or Pacific Islander | 17 (13.7%)  2 (1.6%)  2 (1.6%)  34 (27.4%)  57 (46.0%)  6 (4.8%)  3 (2.4%) |
| Religion, Number (%)  Atheist/Agnostic  Buddhist  Christian  Hindu  Jewish  Muslim  Other | 30 (24.2%)  2 (1.6%)  49 (39.5%)  5 (4.0%)  3 (2.4%)  2 (1.6%)  17 (13.7%) |
| Social Political View, Number (%)  Strongly Conservative  Slightly Conservative  Neutral  Slightly Liberal  Strongly Liberal | 3 (2.4%)  10 (8.1%)  13 (10.5%)  30 (24.2%)  51 (41.1%) |
| Economic Political View, Number (%)  Strongly Conservative  Slightly Conservative  Neutral  Slightly Liberal  Strongly Liberal | 5 (4.0%)  14 (11.3%)  25 (20.2%)  45 (36.3%)  19 (15.3%) |
| Sexual Identity, Number (%)  Bisexual  Heterosexual  Homosexual/Gay/Lesbian  Queer  Other | 2 (1.6%)  101 (81.5%)  1 (0.8%)  2 (1.6%)  2 (1.6%) |
| Profession, Number (%)  Medicine  Nursing  Law  Other | 68 (54.8%)  19 (15.3%)  1 (0.8%)  23 (18.5%) |
| Highest Level of Education  High School  Associates  Bachelors  Masters  Doctorate  Multiple Doctorate | 3 (2.4%)  5 (4.0%)  73 (58.9%)  15 (12.1%)  10 (8.1%)  2 (1.6%) |
| Past Attendee | 33 (30.6%) |
